# A RESPONSE to anti**–**IL-5 therapy in comorbid patients with chronic rhinosinusitis with nasal polyps and severe asthma: Study protocol

**DOI:** 10.1016/j.jacig.2024.100343

**Published:** 2024-09-17

**Authors:** Petros Bakakos, Isam Alobid, Jannis Constantinidis, Peter Hellings, Oliver Pfaar, Camille Taillé, David Bañas-Conejero, Konstantina Kallinikou, Peter Howarth, Florence Schleich

**Affiliations:** a 1st University Department of Respiratory Medicine, National and Kapodistrian University of Athens, Athens, Greece; b Rhinology and Skull Base Unit, ENT Department, Hospital Clínic, Universitat de Barcelona, CIBERES, IDIBAPS, Barcelona, Spain; c Aristotle University, Thessaloniki, Greece; d Department of Otorhinolaryngology, University Hospitals Leuven, Leuven, Belgium; e Laboratory of Allergy and Clinical Immunology, University of Leuven, Leuven, Belgium; f Upper Airways Research Laboratory, University of Ghent, Ghent, Belgium; g Department of Otorhinolaryngology, Head and Neck Surgery, Section of Rhinology and Allergy, University Hospital Marburg, Philipps-Universität Marburg, Marburg, Germany; h Department of Respiratory Diseases, Bichat Hospital, AP-HP Nord-Université Paris Cité, UMR 1152, Paris, France; i Specialty Medical Department, GSK, Tres Cantos, Spain; j Europe Medical, Specialty Care, GSK, Athens, Greece; k Global Medical, Specialty & Primary Care, GSK, London, United Kingdom; l CHU Sart-Tilman Liege, B35, University of Liege, GIGA I3, Liege, Belgium

**Keywords:** Chronic rhinosinusitis with nasal polyps, nasal polyps, asthma, severe asthma, comorbidity, T2 inflammation, SNOT-22, real world

## Abstract

**Background:**

Chronic rhinosinusitis with nasal polyps (CRSwNP) and severe asthma (SA) are 2 frequently coexisting conditions that are, in most cases, associated with eosinophilic inflammation. The concurrence of both diseases has a negative synergistic impact on disease severity and patients’ health-related quality of life. Thus, a holistic, collaborative management of these patients is a critical unmet need. Mepolizumab, a systemic anti–IL-5 therapy, has been shown to be effective as an add-on treatment in both SA and CRSwNP, with more literature available on asthma outcomes than on CRSwNP.

**Objectives:**

The primary objective of the study is to evaluate the real-world effectiveness of mepolizumab in improving the health-related quality of life of comorbid patients at 12 months using the SNOT-22 questionnaire. Secondary objectives include safety and efficacy outcomes of mepolizumab treatment in the 2 populations, which are expected to have variable severity of the respective comorbid conditions.

**Methods:**

RESPONSE is a European real-world prospective cohort study designed to assess the effectiveness of mepolizumab in 2 cohorts of adult patients: one with SA as primary diagnosis with (secondary diagnosis) comorbid CRSwNP, and another with CRSwNP as primary diagnosis with (secondary diagnosis) comorbid asthma. Up to 350 patients receiving newly prescribed mepolizumab will be followed up for 12 months as per the investigators’ standard of care.

**Conclusion:**

This study will report the effects of anti–IL-5 therapy in both diseases investigated and the respective comorbidity, as well as the consequence of treating milder forms of asthma and CRSwNP with mepolizumab, supporting the emerging evidence on early treatment optimization.

Asthma and chronic rhinosinusitis with nasal polyps (CRSwNP) are 2 prevalent and frequently associated chronic inflammatory diseases that impose a significant clinical burden on affected patients.^1^^,^^2^ Up to 65% of patients with CRSwNP have comorbid asthma,^3^ and up to 40% of patients with severe late-onset asthma have comorbid CRSwNP.^4^ Their common coexistence reflects their common pathophysiology, with tissue eosinophilic inflammation being the norm in CRSwNP,^3^ and with over 80% of patients with severe asthma (SA) having an eosinophilic phenotype (SA-EP).^5^^,^^6^ IL-5 is a key driver of eosinophilic inflammation, systemically signaling the bone marrow to enhance eosinophil maturation, activation, and survival.^7^ Additionally, IL-5 has been shown to affect other cell types such as epithelial cells, disrupting the upper and lower airway epithelial barrier integrity.^8^^,^^9^ Moreover, its presence in polyp tissue after endoscopic sinus surgery is associated with greater rates of nasal polyp (NP) recurrence.^10^^,^^11^ As such, type 2 (T2)-associated disease throughout the airways contributes to eosinophilic inflammation and patients with comorbid upper and lower airway disease (CRSwNP and asthma) have higher blood eosinophil counts than either disease in isolation.^12^^,^^13^ Furthermore, it has been shown that stimulated eosinophilic inflammation at one airway site worsens eosinophilic inflammation at the other airway site.^14^ Indeed, the presence of comorbid SA-EP substantially increases the probability that a patient’s NPs will be eosinophilic.^12^ There is thus, a close inflammatory association between asthma and CRSwNP, in which eosinophils and IL-5 play a central role. A recent study highlighted another feature that links these 2 diseases, namely the presence of specific IgE against *Staphylococcus aureus* enterotoxins.^15^ Patients with asthma positive for *S aureus* enterotoxin IgE were found to be more likely to have comorbid CRSwNP than those without. These patients showed a higher rate of exacerbations, more severe airway obstruction, and higher levels of airway and serum T2 biomarkers.^15^

This is because the presence of comorbid disease, whether that be asthma in CRSwNP or CRSwNP in asthma, worsens disease outcomes at the other site. As such, the presence of CRSwNP in asthma patients is generally associated with an increased likelihood of severe exacerbations, a reduced likelihood of asthma control requiring more therapy with oral corticosteroids (OCSs), and worse lung function.^16^, ^17^, ^18^, ^19^ Moreover, asthma and comorbid CRSwNP have a negative impact on health-related quality of life (HRQoL), including physical and mental health, social functioning, sleep, and workplace absenteeism.^20^^,^^21^ Similarly, the presence of asthma in CRSwNP is associated with worse outcomes, as reflected by the increased need for sinus surgery, along with greater postsurgical recurrence as well as the need for more frequent OCS bursts.^22^, ^23^, ^24^, ^25^, ^26^ Thus, the appropriate management of these patients, in a holistic manner, including close collaboration between ear, nose, and throat specialists, allergists, and pulmonologists, covering both their upper and lower airways with safe systemic therapy, is a critical unmet need and an important clinical target for health care providers.

Mepolizumab is a humanized anti–IL-5 monoclonal antibody used for the treatment of SA since 2015.^27^^,^^28^ A comprehensive clinical development program has demonstrated its efficacy in reducing asthma exacerbations and OCS receipt, as well as in improving lung function and overall HRQoL in SA. Consistent with randomized controlled trials (RCTs), recent real-world evidence conducted in patients with SA, indicates that mepolizumab is effective in the wider patient profile seen in clinical practice.^18^^,^^29^, ^30^, ^31^, ^32^, ^33^, ^34^

In 2021, mepolizumab was additionally authorized for the treatment of adult patients with CRSwNP for whom therapy with systemic corticosteroids and/or surgery do not provide adequate disease control.^28^ The authorization was based on a randomized, double-blind, placebo-controlled, parallel-group, phase 3 trial of patients with severe bilateral CRSwNP (SYNAPSE), which found that mepolizumab treatment improved NP size and obstruction compared to placebo.^35^ Consequently, mepolizumab has been included in international guidelines for CRSwNP’s standard of care, including the European Position Paper on Rhinosinusitis, the International Consensus Statement on Allergy and Rhinology, the European Forum for Research and Education in Allergy and Airway Diseases, and the recently published guidelines of the German Society of Oto-rhino-laryngology, Head and Neck Surgery (DGHNO), and the German College of General Practitioners and Family Physicians (DEGAM), and is the first anti–IL-5 biologic treatment for severe uncontrolled CRSwNP approved in Europe.^20^^,^^36^, ^37^, ^38^

Evidence for the effect of mepolizumab on patients with comorbid CRSwNP and asthma comes from subanalyses of RCT studies (SYNAPSE, MENSA, and MUSCA studies).^13^^,^^35^^,^^39^^,^^40^ These studies show the benefit of systemic therapy with mepolizumab in improving disease outcome at both airway sites.^13^^,^^41^ Although the effectiveness of mepolizumab in improving asthma HRQoL for comorbid patients in clinical practice is well documented,^42^, ^43^, ^44^ there is limited prospective evidence on its effectiveness in patients with CRSwNP in real-world settings, or on CRSwNP-related outcomes in patients with CRSwNP and comorbid asthma. This real-world study will provide an in-depth analysis of the upper airway benefits associated with mepolizumab use in comorbid patients.

This study aims to provide insight into the effectiveness of mepolizumab in 2 cohorts of patients, one with SA as primary diagnosis with comorbid CRSwNP of any severity, and another with CRSwNP as primary diagnosis with comorbid asthma of any severity, examining both NP- and asthma-related outcomes with a focus on the upper airway symptoms. The results will provide a better understanding of the similarities and differences between these 2 cohorts in their disease characteristics and clinical profile, as well as provide information about the clinical effectiveness and safety of mepolizumab in real-world settings, as well as insight into the predictors of response. Through the dual indication entry criteria and the aforementioned overlap between these 2 comorbidities, we will be able to explore the effects of IL-5 inhibition earlier in the course of both diseases and evaluate potential benefits for the patients in a prospective real-world scenario.

## Methods

### Study design and patients

**Fig 1:**
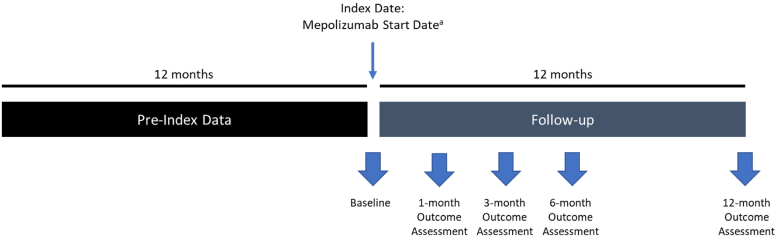
Study schematic. ^a^Mepolizumab start date/baseline could be up to 30 days before or after baseline visit.

RESPONSE is a multinational real-world prospective cohort study designed to assess the effectiveness of mepolizumab in adult patients with comorbid asthma and CRSwNP. Patients from otorhinolaryngology, pulmonology, and allergy services will be consecutively enrolled in Austria, Belgium, France, Germany, Greece, Italy, the Netherlands, Spain, and the United Kingdom. Patients’ demographic and clinical features will be documented at baseline and followed up for 12 months during the mepolizumab treatment, with 4 estimated visits at approximately 1, 3, 6, and 12 months as determined by their health care providers’ routine standard of care (Fig 1).

Key inclusion criteria are a primary diagnosis of SA-EP and comorbid CRSwNP, or a primary diagnosis of severe uncontrolled CRSwNP with comorbid asthma as defined by the prescribing physician. Further inclusion criteria include: having valid Sinonasal Outcome Test 22 (SNOT-22) and visual analog scale (smell and nasal obstruction), and a prescription of mepolizumab 0 to 30 days before study enrollment. Key exclusion criteria are current participation in other investigational studies and having had recent NP surgery (<6 months before enrollment), or surgery scheduled to occur within 1 month after the enrollment visit. A detailed list of inclusion and exclusion criteria is available in Table E1 in this article’s Online Repository available at www.jaci-global.org. The study will be carried out in line with the principles of the Declaration of Helsinki and will follow central and local approval via hospitals institutional review boards.

### Outcomes

The primary objective of this study is to evaluate the real-world effectiveness of mepolizumab in improving the HRQoL of comorbid patients at 12 months, assessed by the SNOT-22 questionnaire. Secondary outcomes aim to describe the demographic and clinical features of patients with comorbid upper and lower airway disease, and to evaluate the effectiveness of mepolizumab in reducing asthma exacerbations, and OCS receipt (which will also be evaluated as a confounding factor in the analysis plan). Other key secondary objectives are the assessment of lung function (forced expiratory volume in 1 second) and disease control and quality of life outcomes (Asthma Control Questionnaire 5 [ACQ-5] and Asthma Quality of Life Questionnaire [AQLQ] scores), as well as the effect on a series of biomarkers (blood eosinophil counts, fractional exhaled nitric oxide [Feno], IgE). Safety and tolerability will be followed up by the reporting of drug-related adverse events. The detailed objectives and end points of the study are shown in Table I.

**Table I:** Summary of RESPONSE study end points

Objective	Description	End point
Primary efficacy	• Effectiveness at 1 year of mepolizumab in upper airway disease–related QoL	• Change in SNOT-22 score at 12 months
Key secondary NPs	• Short-term effectiveness of mepolizumab in upper airway disease–related QoL • Effectiveness of mepolizumab in improving smell dysfunction • Effectiveness of mepolizumab in improving nasal obstruction • Effectiveness of mepolizumab in reducing size of NPs	• Change from baseline in SNOT-22 score at 1, 3, and 6 months • Change from baseline in VAS score for smell dysfunction at 1, 3, 6, and 12 months • Change from baseline in VAS score for nasal obstruction at 1, 3, 6, and 12 months • NP score before mepolizumab treatment and at 1, 3, 6, and 12 months as evaluated by nasal endoscopy and/or CT scan in patients with NPs at baseline
Key secondary asthma	• Evaluate effectiveness of mepolizumab in reducing asthma exacerbations of comorbid patients • Assess mepolizumab effectiveness in improving asthma control in comorbid patients • Evaluate capacity to improve asthma QoL of mepolizumab in real-world setting in comorbid patients	• Change in clinically significant asthma exacerbation rate in patients at 12 months compared to rate in 12-month premepolizumab treatment period • ACQ-5 change at 1, 3, 6, and 12 months vs baseline • AQLQ change at 1, 3, 6, and 12 months vs baseline
Secondary efficacy end points pertaining to treatments received	• Describe mepolizumab treatment patterns over follow-up period for patients receiving mepolizumab, including but not limited to dose modifications, treatment cessation, changes in concomitant medications or procedures • Assess effectiveness of mepolizumab in tapering OCS receipt in comorbid patients	• Start date, dose, frequency, cessation, changes, and interruption of mepolizumab and other concomitant treatments for asthma or CRSwNP • Change in OCS daily dose utilization at 1, 3, 6, and 12 months vs baseline dose
Safety end points	• Describe real-world safety associated with mepolizumab in comorbid patients	• Incidence of adverse events related to mepolizumab during study
Exploratory	• Measure capacity of mepolizumab to result in clinical disease remission of comorbid patients • Evaluate if there is a change in Feno with mepolizumab treatment of comorbid patients in real-world setting • Evaluate change in FEV_1_ with mepolizumab treatment of comorbid patients in real-world setting • Evaluate change in blood eosinophil levels with mepolizumab treatment of comorbid patients in real-world setting • Describe results of all study objectives separately in each of 2 study cohorts	• Proportion of patients with disease in clinical remission • Change in Feno, when available, at 1, 3, 6, and 12 months vs baseline • Change in FEV_1_, when available, at 1, 3, 6, and 12 months vs baseline • Change in blood eosinophil levels, when available, at 1, 3, 6, and 12 months vs baseline • Change in previous end points in each study cohort

*ACQ-5,* Five-question Asthma Control Questionnaire; *AQLQ,* Asthma Quality of Life Questionnaire; *CT,* computed tomography; *FEV*_*1*_*,* forced expiratory volume in 1 second; *QoL,* quality of life; *VAS,* visual analog scale.

### Sample size

Approximately 350 patients (accounting for an approximately 20% dropout rate over the course of the study) will be enrolled from several European countries. Sample size calculations were performed to ensure significant statistical precision to measure a change from baseline in SNOT-22 (Table II).

**Table II:** Sample size and precision of paired mean assuming alternative sample size scenarios and fixed SD of 17

Sample size∗	Precision†	95% CI of change‡		
		9 units	10 units	11 units
150	2.72	6.3, 11.7	7.3, 12.7	8.3, 13.7
200	2.36	6.6, 11.4	7.6, 12.4	8.6, 13.4
250	2.11	6.9, 11.1	7.9, 12.1	8.9, 13.1
300	1.92	7.1, 10.9	8.1, 11.9	9.1, 12.9
350	1.78	7.2, 10.8	8.2, 11.8	9.2, 12.8

*CI,* Confidence interval; *SD,* standard deviation.

∗ At least half of patients enrolled should have CRSwNP with comorbid asthma as primary diagnosis.

† Distance from observed mean (ie, mean change from baseline) to upper/lower 95% CI limit.

‡ Expected 95% CI assuming 9-, 10-, and 11-unit change from baseline. CI width is expected to be same as SD of difference, 17, and thus has been used.

### Statistical analysis

The primary analysis of the change from baseline in SNOT-22 will use mixed effects models for repeated measures by including time as a categorical variable, when appropriate. Descriptive analysis of the data will be performed using summary statistics for categorical and quantitative (continuous) data. Frequency tables will be generated for categorical data. Counts of missing data will be provided in all tables for information only. Percentages will not include the missing category and will be calculated from the number of patients with available (nonmissing) data. Continuous data will be described by the number of nonmissing values, median, mean, standard deviation, minimum, and maximum as well as lower and upper quartiles.

## Discussion

Upper and lower airways disease commonly coexist in patients who seek clinical care with SA or CRSwNP. These diseases share a common underlying pathophysiology, with elevated IL-5 and eosinophilic inflammation being evident in both, compared to that in health,^45^^,^^46^ as well as common trigger factors, such as aspirin and nonsteroidal anti-inflammatory therapy in those with aspirin-exacerbated respiratory disease, otherwise termed nonsteroidal therapy–exacerbated respiratory disease.^47^^,^^48^ It is thus a logical consideration that a systemic therapy that targets the underlying disease process would benefit patients with comorbid disease in improving outcomes at both airway sites. In this respect, it is well recognized that OCSs, as a systemic therapy, do indeed improve outcomes for both asthma and CRSwNP but are limited by their short-term and a long-term systemic toxicity.^49^ Mepolizumab, as a subcutaneously administered anti–IL-5 therapy, is a precision-targeted systemic therapy directed against the underlying disease process in both CRSwNP and SA, with a favorable safety profile compared to OCS.^35^^,^^49^ Mepolizumab has been shown in RCTs to improve disease control in SA and in CRSwNP.^35^^,^^39^^,^^40^ There were patients with comorbid airways disease in these studies, and analysis identified improvements in upper airway outcomes in those treated for SA and lower airways disease outcomes in those treated for CRSwNP.^13^^,^^41^ However, the assessments of comorbid disease outcomes were limited because that was not the primary intention of these disease-targeted RCTs. The RESPONSE study has thus been established to extend these findings in a more comprehensive prospective manner, focusing only on patients with comorbid upper and lower airway disease.

To our knowledge, this study is unique in that it will not focus solely on either SA with comorbid CRSwNP or on CRSwNP with asthma, but rather will recruit patients for whom clinicians have independently decided to prescribe mepolizumab to treat an airway disease, whether SA or CRSwNP be the primary indication. The requirement is, however, that there is a history of comorbid airway disease. Both asthma and CRSwNP impair quality of life, with greater impairment when both diseases coexist.^50^ In CRSwNP, there is greater bodily pain and reduced physical functioning and vitality if there is comorbid asthma.^51^ Furthermore, in CRSwNP, the presence of persistent asthma has an accumulative impact on the loss of smell,^52^^,^^53^ potentially related to a more intense tissue eosinophilic inflammation because tissue eosinophilia is linked to impaired sense of smell.^54^^,^^55^ Consistent with this, there is a greater drive to eosinophilia in comorbid disease, with blood eosinophil measures being higher in patients with comorbid asthma and CRSwNP than either disease alone.^12^ Similarly, those with SA who have comorbid CRSwNP have worse quality of life than those with SA alone.^13^^,^^56^ The presence of CRSwNP as a comorbidity with SA is associated with worse asthma control, more frequent SA exacerbations, and a greater likelihood of escalation to maintenance oral steroid therapy.^16^^,^^24^, ^25^, ^26^ As a result of the adverse impact of comorbid disease on quality of life, SNOT-22 questionnaire results, as an integrated evaluation of the comorbid disease impact, have been selected as the key patient-reported outcome in this study, and the impact of mepolizumab on SNOT-22 as the primary study outcome.

The design of the study, focusing on patients with a primary diagnosis of SA or CRSwNP who have airway disease comorbidity, will allow an assessment of the impact of treatment on the respective comorbid condition within a population with heterogeneous severity levels. It is recognized that while asthma is a common comorbidity in CRSwNP, there is a spectrum of disease severity; many will not have SA. This will thus allow evaluation of the impact of mepolizumab in an asthma population before progression to severe disease. Similarly, those with CRSwNP in the SA-treated cohort will have a range of disease severity and are likely to include patients who may not have had previous surgery, unlike the population in the mepolizumab pivotal CRSwNP RCT, in which all subjects had undergone previous surgery, often multiple, and were being listed for further surgery on account of troublesome disease recurrence. Furthermore, the authors expect that there will be patients whose NPs are at an earlier stage of progression, not necessarily grade 3-4. The levels of tissue eosinophils and IL-5 levels in removed polyps is a predictive factor for polyp recurrence,^8^ so polyps caught earlier in their development may be more amenable to resolution with anti–IL-5 therapy than those whose growth is already substantial, and this study should be able to evaluate that.

The strength of this prospective study is that there is an objective evaluation of polyp size before and at several points during the follow-up period on mepolizumab with nasal endoscopy or computed tomographic scan imaging, as per investigators’ routine clinical practice. One of the limitations of the previous RCT studies of SA was that the diagnosis of CRSwNP was an historic one, and no true assessment of polyp presence was undertaken. In the RESPONSE study, all participants will have their polyp status documented at entry. For those treated for CRSwNP, biomarker measures will be followed (Feno and blood eosinophils), as well as patient-reported outcomes, such as those assessed by the ACQ, the AQLQ, and serial measures of lung function that will provide greater insight into the impact of mepolizumab on asthma and how this relates to disease phenotype and baseline characteristics. This will support emerging evidence on the potential for better clinical remission outcomes with treatment earlier in the course of asthma while lung function is still relatively well preserved and symptoms are not too burdensome.^57^

The RESPONSE study will reinforce, with prospective real-world data, the existing evidence from retrospective studies that have shown significant improvement after mepolizumab treatment mostly in patients with either CRSwNP or SA-EP.^58^, ^59^, ^60^

Other strengths of this study are the prospective nature of data collection, the multicentric and international scope, and the fact that most variables and analyses have been designed with routine clinical practice as a reference, thereby minimizing the risk of missing data—which is still a relevant limitation of this study because medical records will be the primary source of information, especially for baseline and pretreatment records. The inclusion of data after the first month will be useful to evaluate how soon both conditions improve after initiation of mepolizumab therapy.

In summary, the RESPONSE study is designed to deliver new insights on the impact of anti–IL-5 therapy with mepolizumab in T2 concomitant upper and lower airway inflammation and will evaluate the potential benefits of timely treatment optimization in these patients.

## Disclosure statement

The RESPONSE study is funded by GSK (study ID 218616).

Disclosure of potential conflict of interest: F. Schleich reports consultancy fees from GSK, AstraZeneca, Novartis, and Chiesi; and grants from GSK, AZ, and Chiesi; and honoraria from GSK as part of involvement in this study’s design. P. Hellings reports consultancy, lecture, and/or research grants from GSK, 10.13039/100004339Sanofi/10.13039/100004336Novartis, ALK, Stallergenes, and Viatris; and honoraria from GSK as part of involvement in this study’s design. J. Constantinidis reports honoraria for consultancy from GSK; and honoraria from GSK as part of involvement in this study’s design. P. Bakakos reports consultancy fees from GSK, AstraZeneca, Chiesi, Pfizer, ELPEN, and Menarini; and honoraria from GSK as part of involvement in this study’s design. I. Alobid reports honoraria for consultancy and conferences from AstraZeneca, Viatris, Roche, Sanofi, GSK, MSD, Menarini, Salvat, Olympus, and Novartis; and honoraria from GSK as part of involvement in this study’s design. O. Pfaar reports grants and/or personal fees from ALK-Abelló, 10.13039/100009946Allergopharma, Stallergenes Greer, HAL Allergy Holding BV/HAL Allergie, Bencard Allergie/Allergy Therapeutics, Lofarma, ASIT Biotech Tools, Laboratorios LETI/LETI Pharma, GlaxoSmithKline, ROXALL Medizin, Novartis, Sanofi-Aventis and Sanofi-Genzyme, Med Update Europe, streamedup!, Pohl-Boskamp, Inmunotek, John Wiley and Sons, AS, Paul-Martini-Stiftung (PMS), Regeneron Pharmaceuticals, RG Aerztefortbildung, Institut für Disease Management, Springer, AstraZeneca, IQVIA Commercial, Ingress Health, Wort&Bild Verlag, Verlag ME, Procter&Gamble, ALTAMIRA, Meinhardt Congress, Deutsche Forschungsgemeinschaft, Thieme, Deutsche AllergieLiga, AeDA, Alfried-Krupp Krankenhaus, Red Maple Trials, Königlich Dänisches Generalkonsulat, Medizinische Hochschule Hannover, ECM Expro & Conference Management, and Technical University Dresden (all outside the submitted work and within the last 36 months); membership in EAACI Excom and member of external board of directors for DGAKI; and coordinator, main author, or coauthor of different position papers and guidelines in rhinology, allergology, and allergen-immunotherapy; and honoraria from GSK as part of involvement in this study’s design. C. Taillé reports lecture or advisory board fees and grants from AstraZeneca, Sanofi, GSK, Chiesi, and Novartis; and honoraria from GSK as part of involvement in this study’s design. D. Bañas Conejero, K. Kallinikou, and P. Howarth are GSK employees and hold GSK stocks/shares.

## Supplementary data

Supplementary Table E1
