# Supplementary material for: A RESPONSE to anti–IL-5 therapy in comorbid patients with chronic rhinosinusitis with nasal polyps and severe asthma: Study protocol
Source: J Allergy Clin Immunol Glob. 2024 Sep 17;4(1):100343. doi: 10.1016/j.jacig.2024.100343 (PMC11567123; doi:10.1016/j.jacig.2024.100343)
Supplement: Supplementary Table E1 [file mmc1.docx]

# A RESPONSE to comorbid patients – the study protocol

# Supplementary Tables

## Table S1. Complete list of inclusion/exclusion criteria

| **Inclusion criteria** | **Exclusion criteria** |
| --- | --- |
| - Patients with an investigator’s primary diagnosis of SAep with comorbid NP or CRSwNP with comorbid asthma | - Current participation in an interventional study that includes treatment with an investigational drug and/or intervention at the same time as enrolment in the current study or within 6 months prior to initiating mepolizumab |
| - Aged ≥18 years | - Pregnant or breastfeeding at the enrolment visit* |
| - Newly initiating add-on mepolizumab defined as 1) initiating mepolizumab up to 30 days before study enrolment (provided SNOT-22 and VAS scores for smell dysfunction and nasal obstruction has been collected 0 to 30 days before mepolizumab initiation) or 2) initiating mepolizumab up to 30 days after the study enrolment, (provided the decision to prescribe was documented prior to enrolment) | - Diagnosis of eosinophilic granulomatosis with polyangiitis, cystic fibrosis, immunodeficiency, and primary ciliary dyskinesia, or any other condition that would make it difficult for the participant to fulfil the study criteria, per the investigator’s discretion |
| - Eligible for mepolizumab per local labelling requirements | - NP surgery scheduled to occur within 1 month after the enrolment visit |
| - Written informed consent | - NP surgery in the 6 months prior to the enrolment visit |
| - Patient must have access to a compatible device and internet access for ePRO completion and be willing and able to comply with ePRO data collection |  |

CRSwNP, chronic rhinosinusitis with nasal polyps; SNOT-22, Sino-Nasal Outcome test with 22 questions, SAep: severe asthma with eosinophilic phenotype

* Patients that get pregnant during the study will not be excluded if they consent to continue with the study medication.
